# Clinical Characteristics and Long-Term Effects of COVID-19 in a Secondary Care Hospital in South India: A Study of Post-acute Sequelae and Pulmonary Outcomes

**DOI:** 10.7759/cureus.85576

**Published:** 2025-06-08

**Authors:** Lina James, Anil M Philip, Jim O John, Ayobami G Olafimihan, Fernando Esparza

**Affiliations:** 1 Internal Medicine, John H. Stroger, Jr. Hospital of Cook County, Chicago, USA; 2 Internal Medicine, Kuriakose Chavara Memorial Hospital, Nooranad, IND; 3 Internal Medicine, Mitera Hospital, Kottayam, IND; 4 Primary Care/Geriatrics, Oak Street Health, Chicago, USA

**Keywords:** covid-19, long covid symptoms, post-acute sars-cov-2, post-covid functional status, pulmonary function

## Abstract

The coronavirus disease 2019 (COVID-19) pandemic has resulted in significant global morbidity, with long-term effects known as post-acute sequelae of SARS-CoV-2 (PASC) or long COVID syndrome. This study investigates the clinical characteristics and long-term effects of COVID-19 in a rural South Indian population. Conducted in a secondary healthcare setting in Kerala, India, the study involved 65 subjects who had tested positive for COVID-19 more than 90 days prior. Participants completed a detailed questionnaire on common COVID-19 and PASC symptoms and underwent pulmonary function testing. Data analysis included descriptive statistics, point-biserial correlation, and logistic regression. The prevalence of PASC symptoms was found to be 55.4%, with dyspnea being the most common symptom. Healthcare workers experienced a lower prevalence of PASC (41.9%) compared to non-healthcare workers (67.6%). Logistic regression indicated higher odds of PASC in men, non-healthcare workers, and those with comorbidities, though these findings were not statistically significant. Pulmonary function tests revealed reduced forced vital capacity in 23.1% of subjects. The study highlights the significant impact of long COVID on this unvaccinated population prior to the Delta wave, emphasizing the need for ongoing research to understand and manage the long-term effects of COVID-19.

## Introduction

Coronavirus disease 2019 (COVID-19) has triggered an unprecedented global health crisis, affecting more than 750 million people worldwide. In response, several studies have examined the long-term consequences of COVID-19, commonly referred to as post-acute sequelae of SARS-CoV-2 (PASC) or long COVID syndrome. According to the World Health Organization (WHO), PASC is defined as a condition occurring in individuals with a history of probable or confirmed SARS-CoV-2 infection, usually three months after the onset of COVID-19, with symptoms lasting at least two months and not explained by an alternative diagnosis [1]. An international survey from 56 countries recorded 203 symptoms of PASC, with 66 persisting for more than seven months [2]. The Centers for Disease Control and Prevention (CDC) recently reported that one in 13 adults in the United States experiences symptoms of PASC, with the incidence being higher among adults in their 60s [3]. The objectives of our study were to estimate the prevalence of long COVID symptoms persisting for more than 12 weeks; identify the most common ongoing symptoms and their impact on daily functioning; examine variations in symptom patterns based on age, sex, and healthcare worker status; explore links between comorbidities and the presence of post-COVID symptoms; and investigate the relationship between long COVID symptoms and pulmonary function abnormalities.

## Materials and methods

This observational study was conducted in a secondary care setting in rural Kerala (St. Thomas Mission Hospital, Kattanam, India). The primary objective was to estimate the prevalence and impact of post-acute COVID-19 syndrome (PASC), defined as one or more persistent symptoms lasting beyond 12 weeks following confirmed SARS-CoV-2 infection, based on the WHO definition at the time.

To ensure representativeness, multiple recruitment strategies were employed. Individuals who had tested positive for SARS-CoV-2 more than 90 days prior were identified through hospital laboratory records. These included both patients and healthcare workers. They were contacted via telephone and invited to participate in the study. In addition to patients visiting the outpatient clinic, their accompanying relatives with a confirmed history of COVID-19 > 90 days were recruited from outpatient departments over three consecutive days in February 2021, thereby broadening the community representation. Given the exploratory nature of this study and the constraints of conducting research in a rural setting during the early phase of the pandemic, a convenience sampling strategy was employed based on patients accessible within a predefined recruitment window. As such, the sample size was not determined by formal power calculations. While this limits the generalizability of the findings, the study provides important preliminary insights and highlights the need for larger, adequately powered studies to confirm these observations.

Informed consent was obtained at the time of enrolment. Participants completed a structured questionnaire capturing commonly reported symptoms of acute and post-acute COVID-19, based on global clinical data and literature at the time of recruitment. The questionnaire was available in both English and Malayalam. Respondents could fill out the questionnaire independently or with the help of a study investigator to minimize language-related selection bias. Functional status was assessed using the WHO Post-COVID Functional Status (WHO PCFS) scale, evaluating limitations in daily activities due to persistent symptoms.

All participants underwent pulmonary function testing using the Spirolab MIR desktop spirometer (MIR, Italy). The device was calibrated daily per the manufacturer's specifications. Testing was conducted by a certified respiratory technician following the American Thoracic Society (ATS)/European Respiratory Society (ERS) 2019 technical standards for spirometry performance. Parameters measured included the forced vital capacity (FVC), forced expiratory volume in one second (FEV₁), FEV₁/FVC ratio, and peak expiratory flow rate (PEFR). A minimum of three acceptable and reproducible efforts were recorded per participant, with the best effort selected for analysis. Pulmonary function results were initially reported by the technician and subsequently verified by a pulmonologist to be following the 2019 ATS/ERS Technical Standard for Interpretative Strategies in Spirometry, to ensure accurate and standardized interpretation.

Descriptive statistics were used to summarize the demographic and clinical characteristics of the study population. Categorical variables, such as sex, presence of comorbidities, and symptom prevalence, were expressed as frequencies and percentages. Continuous variables, including age and spirometric measurements, were presented as means with standard deviations. To assess the association between age and the presence of post-acute COVID-19 syndrome (PASC) symptoms, a point-biserial correlation was used. Bivariate comparisons were made to explore gender differences in symptom prevalence, using proportions. Differences in PASC symptom rates between healthcare and non-healthcare workers were also described and interpreted in light of age differences across the groups. To examine the independent predictors of post-COVID symptoms, a logistic regression model was built, incorporating covariates such as age, sex, healthcare worker status, and the presence of comorbidities.

Due to the limited sample size, results were interpreted with caution, especially in cases of non-significant findings. Pulmonary function parameters-specifically, FVC and FEV₁-were analyzed to identify patterns of ventilatory impairment. The prevalence of reduced FVC (<80%) was reported. To explore the relationship between acute-phase treatment and pulmonary outcomes, patients were grouped based on whether they received oral azithromycin. After excluding two patients who received additional overlapping therapies, the two treatment groups were matched for age and sex. An independent samples t-test was used to compare mean FVC values between the groups. A p-value < 0.05 was considered statistically significant. All analyses were conducted using R version 4.3.1 (R Core Team, 2023).

## Results

In our study, a total of 65 subjects were enrolled, comprising 40 women and 26 men, with an M:F ratio of 5:8. The average age of participants was 36.2 ± 16 years. Among them, 64.6% had no known comorbid conditions, while diabetes mellitus, systemic hypertension, and dyslipidemia each had a prevalence of 18.5%. Bronchial asthma and coronary artery disease were observed in 7.7% of patients, and hypothyroidism in 3.1%. Additionally, 15.4% and 26.2% of patients were exposed to cigarette smoke or indoor biomass fuel, respectively.

Regarding initial COVID-19 symptoms, fever was the most common, affecting 43.1% of patients. Other prevalent symptoms included headaches (35.4%), anosmia (32.3%), coryza (26.2%), general myalgia (23.1%), fatigue (21.5%), and dysgeusia (20%). Respiratory complaints, such as dyspnea (15.4%) and cough (13.8%), were also observed. During this period, 16.9% of patients were admitted to community isolation centers, with an average admission duration of 8.6 days. None of the patients required oxygen supplementation or ICU admission.

Among the treated patients, 80% received one of the available treatments, including zinc and vitamin C supplements (48 patients), azithromycin (21 patients), ivermectin (two patients), hydroxychloroquine (one patient), and oral prednisolone (one patient). In our study, 55.4% of patients experienced long COVID symptoms beyond 90 days. Dyspnea was the most common (60%), followed by cough (33.3%), fatigue (20%), and insomnia (20%). All patients reported limitations in daily activities due to these lingering symptoms, as indicated by a PCFS score of ≥1.

Gender differences in PASC symptoms were evident, with men presenting a higher prevalence of at least one post-COVID-19 symptom (61.5% vs. 51.2%). Men had higher rates of respiratory symptoms such as cough and dyspnea (57.7% vs. 35.9%), while women had a higher prevalence of insomnia (17.9% vs. 3.8%). Point-biserial correlation analysis showed a weak but statistically significant positive correlation between age and the presence of PASC symptoms (r = 0.34, p = 0.004), with mean ages of 49.2 years for those with symptoms and 34 years for those without.

Nearly half (47.7%) of the study population were healthcare workers, including nurses, lab workers, counselors, and doctors. The prevalence of long COVID syndrome was lower among healthcare personnel compared to non-healthcare individuals (41.9% vs. 67.6%), possibly due to their lower mean age. Logistic regression analysis indicated that men, non-healthcare workers, and individuals with comorbidities might have higher odds of experiencing post-COVID symptoms, although these findings were not statistically significant (p > 0.05).

Lung function assessment revealed a mean FVC of 84.9% ± 16% and a mean FEV1% of 90.9% ± 16.2%. Fifteen subjects (23.1%) had an FVC of less than 80%, suggesting a possible restrictive pathology. However, this could not be further characterized due to the absence of imaging.

Given the large proportion of patients who received azithromycin, a subgroup analysis was done to evaluate the effects of different treatment options during the acute phase of COVID-19. Two patients in the azithromycin group who received additional treatments were excluded. The groups were matched for age and sex (Table 1). The mean FVC was higher in the azithromycin group compared to the non-azithromycin group using the t-test, though this difference was not statistically significant (p = 0.5).

**Table 1:** Demographic and Clinical Characteristics of Patients With and Without Azithromycin Treatment FVC: forced vital capacity; FEV_1_: forced expiratory volume in one second

	Received azithromycin (N = 19)	Did not receive azithromycin (N = 19)
Number of males	11	11
Number of females	8	8
Mean age (years)	37.9	39.5
Mean duration since COVID-19 (days)	115	92
Duration of COVID-19 (days)	11.2	11.8
Patients with cough after COVID-19	4	3
Patients with dyspnea after COVID-19	5	6
Mean FVC%	86.9 ± 16.6	83.4 ± 16.3
Mean FEV_1_%	94.6 ± 17.8	88.4 ± 18.2

## Discussion

Our study aimed to examine the effects of PASC among healthcare workers and the general patient population in a secondary health center in South India following the first wave of COVID-19 and to quantify its impact on the quality of life of our participants. Fever was identified as the most common symptom during the acute phase of the illness, consistent with findings from other studies [4]. The global incidence of PASC is estimated at approximately 75 million, based on a conservative 10% incidence rate among all COVID-19 infections [5]. This is higher than the incidence of post-viral sequelae following acute influenza infection [6]. The COVIDENCE UK study reported that SARS-CoV-2 infection led to significantly higher odds of severe symptoms and a range of issues such as taste/smell problems, light-headedness, hair loss, unusual sweating, racing heart, and memory issues compared to non-COVID-19 acute respiratory infections [7]. The pathophysiology of these symptoms may involve persistent infection, reactivation of latent infections, autoimmune responses, chronic inflammation, or changes in microbiota [8,9]. Additionally, heightened fear and stress during the pandemic contributed to more severe symptoms compared to previous pandemics [10]. Research indicates that severe acute infection, advanced age, increased body mass index, and female sex are risk factors for PASC [11]. However, in our study, we observed a lower incidence of PASC symptoms among women, likely due to the lower mean age in the female population.

Meta-analyses indicate that fatigue, dyspnea, sleep disturbances, and difficulties in concentration are among the most common PASC symptoms. In our study, dyspnea was the most frequently reported symptom, affecting 33.3% of participants. The higher incidence of dyspnea in our study may be correlated with the younger age of participants and a higher likelihood of dysfunctional breathing, as reported by Frésard and colleagues [12]. An Indian Council of Medical Research (ICMR) survey reported a 30.34% prevalence of PASC beyond 12 weeks of hospitalization among healthcare workers, with a higher prevalence of insomnia (36% vs. 32.3%) [13]. The higher prevalence of PASC among medical personnel in our study might be attributed to the smaller sample size. However, comparisons between healthcare workers and non-medical personnel showed increased PASC incidence in the general population (41.9% vs. 67.6%), likely related to the lower mean age among medical personnel (28.4 vs. 43.4 years). A study in Vietnam found a higher incidence of insomnia among healthcare workers (36% vs. 32.3%), while our study reported a lower incidence of insomnia (6.5%) among medical personnel. Increased age and lack of social support were highly correlated with higher insomnia rates [14].

Lung function abnormalities were observed, with 31% of patients showing reduced diffusion capacity at one year post infection and 5% having reduced FVC [15]. The higher percentage of reduced FVC in our study (23.1%) could be due to the smaller sample size. Impairments in lung function were more prevalent in patients with severe initial infections [16]. Imaging studies at six months and one year revealed that ground-glass opacities and pulmonary fibrosis were the most common abnormalities, occurring in 34% and 32% of cases, respectively, in meta-analyses [15]. Most imaging abnormalities in patients with COVID-19 pneumonia showed a favorable course toward resolution, with a study of severe cases reporting complete normalization of pulmonary findings in 58% of patients, with resolution occurring by three months for over half and by six months for the remainder [17].

Our study focused on participants who contracted COVID-19 during the initial outbreak in mid to late 2020, corresponding to the first wave in India. The emergence of the Delta variant in late 2020 led to a surge in cases, marking the beginning of the second wave in March 2021 [18]. A recent study showed that among unvaccinated individuals, the cumulative incidence of PASC decreased from 10.42 events per 100 persons in the pre-Delta era to 7.76 events per 100 persons in the Omicron era. Vaccinated individuals experienced a lower incidence of PASC, with 72% of the decrease during the Omicron era attributed to vaccination [19]. Notably, no studies on post-COVID symptoms were published in India during 2020. An ICMR study conducted in early 2021 in South India using the PCFS scale found that 87% of participants had no functional limitations [20]. The discrepancy between this study and ours, where only 47.7% reported full functional efficiency, could be due to a smaller sample size, differences in viral variants, unvaccinated status, or heightened general fear during the early pandemic.

This study also investigated the effects of oral concomitant therapies, including azithromycin, which was commonly used during the observation period. Azithromycin was administered to 32.3% of our patients, reflecting its widespread prescription aimed at minimizing hospitalizations. A multinational study similarly noted a 28.3% prescription rate for azithromycin during the acute phase [21]. Due to the limited sample size, data on adjuvant therapies could not be effectively interpreted, and no statistical significance was observed. Although studies have shown no benefit of azithromycin during the acute phase [22], there are no large studies demonstrating its effects on PASC symptoms. Currently, 525 trials for long COVID are registered on ClinicalTrials.gov, with 20 funded by the NIH [23]. Recent studies suggest that nirmatrelvir treatment during the acute phase is associated with a lower incidence of PASC [24], and preliminary reports indicate that metformin may offer potential benefits for long COVID [25]. Rehabilitation programs, such as the REGAIN study conducted in the UK, have demonstrated effectiveness in managing chronic diseases and improving health-related quality of life in adults with PASC [26].

Long COVID, now formally coded under ICD-10-CM code U09.9, has emerged through a challenging process due to evolving clinical definitions and understanding of its mechanisms [27]. The use of this code in the United States was delayed nearly two years after the condition was first identified by patients. Ongoing research into the pathophysiology, diagnostic criteria, and therapeutic interventions for long COVID is crucial to improving patient outcomes and quality of life.

Limitation

Since our study was conducted three years ago, the landscape of PASC has continued to evolve. Larger and more robust studies have emerged, providing valuable insights into this complex condition. Despite being conducted during an earlier timeframe, our study offers a valuable historical perspective on PASC in a rural South Indian population following the first wave. These findings can be compared with current data to understand potential changes in PASC presentation or prevalence over time.

Our study identified limitations in generalizability due to the sample size and the potential for selection bias, given that the recruitment methods might have predisposed patients with symptoms to enroll in the study, as well as recall bias, given the time elapsed between infection and data collection; these are significant limitations that may affect the accuracy and reproducibility of symptom reporting, highlighting the ongoing need for larger, geographically diverse studies to understand PASC across different populations. We also recognize that incorporating objective measures like standardized symptom scores and laboratory parameters would have significantly enhanced the data. Autoimmune workup to assess potential immune dysregulation and markers of COVID-19 immunity might have provided crucial clues about the underlying mechanisms of PASC.

Furthermore, our study predated India's vaccination campaign, limiting our ability to evaluate the potential impact of vaccination on PASC. Future studies should incorporate vaccination status as a variable to understand its influence on PASC development and symptom severity. Additionally, analyzing PASC symptoms across different COVID-19 waves, including the second wave with new variants, could reveal critical information regarding how viral variants influence symptomatology during and after infection.

The exclusion of imaging data in patients with severe symptoms or abnormal pulmonary function tests (PFTs) is another recognized limitation. Incorporating chest X-rays or high-resolution computed tomography (HRCT) scans would provide valuable information on the extent and pattern of pulmonary involvement in PASC patients. While the low-resource setting inevitably restricted access to advanced diagnostic tools, limiting comprehensive data collection and extensive follow-up, future studies in similar settings may help close this knowledge gap.

Future studies should focus on larger, geographically diverse populations and incorporate standardized symptom scores, laboratory parameters, and imaging data. Longitudinal research with extended follow-up periods is essential to monitor the progression and resolution of symptoms while evaluating the impact of vaccination, different COVID-19 waves, and potential therapeutic interventions. Investigating the psychosocial aspects, genetic predispositions, and immunological responses in individuals with PASC will provide a holistic view of the condition. This approach will help identify persistent symptoms, the role of comorbidities, and the effectiveness of rehabilitation programs, leading to improved diagnostic criteria, therapeutic interventions, and patient outcomes.

## Conclusions

Despite these limitations, our study effectively captures the initial picture of PASC in rural India, addressing public anxieties during a critical time. By acknowledging these limitations, we aim to pave the way for more robust investigations in the future. Our findings offer a perspective on the prevalence and experiences of PASC after the first wave of COVID-19 within a rural healthcare community. Connecting our results with experiences from diverse regions and timeframes encompassing subsequent waves of the pandemic can help us appreciate the widespread impact of this persistent health concern in the post-pandemic world.
